# Effects of exercise training on skeletal muscle function in patients with mitochondrial myopathy: a systematic review

**DOI:** 10.3389/fspor.2026.1710264

**Published:** 2026-04-09

**Authors:** Qiliang Wan, Yi Ding, Wenduo Liu, Kun Yang

**Affiliations:** 1 Liaoning Institute of Basic Medical Sciences, Shenyang, China; 2 Department of Sports Science, College of Natural Science, Jeonbuk National University, Jeonju, Republic of Korea; 3 College of Physical Education, Beihua University, Jilin, China

**Keywords:** exercise intervention, exercise recommendations, mitochondrial function, mitochondrial myopathy, pathological mechanisms

## Abstract

**Background:**

Mitochondrial myopathy (MM) is a group of rare, progressive muscle disorders characterized by impaired oxidative phosphorylation due to mitochondrial DNA (mtDNA) or nuclear DNA (nDNA) mutations, leading to exercise intolerance, muscle weakness, and metabolic dysfunction. Although exercise is increasingly recognized for its capacity to enhance mitochondrial function and muscle performance, the specific effects of different exercise prescriptions (in terms of modality, intensity, and duration) on MM and their phenotype-specific outcomes remain heterogeneous. This study systematically investigates how various exercise types influence mitochondrial function, muscle performance, and clinical outcomes across MM subtypes.

**Methods:**

Databases including PubMed, Web of Science, Embase, and Scopus were searched from 1990 to September 2025. Clinical trials involving exercise interventions in MM patients were included, with outcomes covering exercise capacity, muscle function, mitochondrial markers, and metabolic indices. Risk of bias was assessed using Revised Cochrane Risk-of-Bias Tool for Randomized Trials (RoB 2) and Risk of Bias in Non-randomized Studies of Interventions (ROBINS-I V2), and methodological quality was appraised with the Mixed Methods Appraisal Tool (MMAT).

**Results:**

Fifteen studies (1 randomized controlled trial and 14 non-randomized trials) including a total of 157 MM patients (sample size per study: 4–20) were analyzed. Moderate-intensity aerobic and resistance exercise consistently improved maximal oxygen uptake (VO_2_ max), maximal workload (W max), muscle strength, and mitochondrial enzyme activity, with no consistent group-level increases observed in creatine kinase (CK) levels or mtDNA mutation burden. Aerobic training enhanced oxidative capacity, phosphocreatine (PCr) recovery, and antioxidant defense, while resistance training improved muscle strength, satellite cell activation, and reduced cytochrome c oxidase (COX)-deficient fibers. Combined regimens yielded additive benefits. Most interventions lasted 8–14 weeks, 3–5 sessions per week. Phenotype-specific responses were evident: patients with large-scale deletions or m.3243A>G mutations showed favorable adaptation, whereas other point mutations or microdeletions displayed variable or adverse responses.

**Conclusion:**

Moderate-intensity, phenotype-specific exercise prescriptions, especially those integrating both aerobic and resistance components, may enhance mitochondrial and muscular function in patients with mitochondrial myopathy while reducing the likelihood of adverse effects. However, larger controlled trials are needed to confirm long-term efficacy and to clarify potential risk profiles.

**Systematic Review Registration:**

https://www.crd.york.ac.uk/PROSPERO/view/CRD420251145502, PROSPERO CRD420251145502.

## 1 Introduction

Mitochondria are the primary organelles responsible for energy production (1). Approximately 90% of the body's energy requirements are met by mitochondria. Their primary function is to synthesize adenosine triphosphate (ATP) by oxidizing nutrients through oxidative phosphorylation (OXPHOS), involving the tricarboxylic acid cycle (TCA) cycle and the electron transport chain (ETC, also known as the respiratory chain), thereby supplying energy to various cells. Mitochondria also play a crucial role in cellular metabolism, calcium regulation, and apoptosis. They possess a unique double-membrane structure and their own genetic material.

Skeletal muscle relies on mitochondria to generate ATP through OXPHOS, providing energy for muscle contraction and sustained activity (2, 3). As a metabolic organelle providing ATP, mitochondria play a vital role in maintaining systemic metabolic homeostasis during daily activities and physical exertion.

When mitochondrial OXPHOS function is impaired, it cannot meet cellular energy demands, leading to metabolic disorders and a range of diseases (4–6). Myopathy is one of the primary manifestations of mitochondrial diseases, with clinical symptoms including exercise intolerance, muscle weakness, and muscle atrophy (7). Mitochondrial myopathy (MM) is primarily caused by mutations in mitochondrial DNA (mtDNA) or nuclear DNA (nDNA), leading to OXPHOS dysfunction (8).

Mutations in either the nDNA or mtDNA genomes within mitochondria can disrupt ETC complexes. The high mutation rate and heteroplasmy of mtDNA lead to variable expression of defective OXPHOS components across tissues. Once the proportion of mutant mtDNA exceeds a critical threshold, mitochondrial ATP synthesis declines and oxidative stress increases, particularly in energy-demanding tissues such as skeletal muscle and the brain (9, 10). Dysfunction of the ETC reduces ATP production and causes premature electron leakage, resulting in excessive reactive oxygen species (ROS) generation (11, 12). The ensuing oxidative stress damages mtDNA, further elevating mutation load and initiating autophagic and apoptotic cascades that impair muscle-cell viability. Additionally, decreased expression of mitochondrial calcium-channel proteins such as VDAC disrupts Ca²^+^ homeostasis (13), producing calcium overload and endoplasmic-reticulum stress that aggravate muscle degeneration (14, 15). These alterations reduce mitochondrial efficiency, forcing skeletal muscle to rely increasingly on anaerobic glycolysis (16).

**Figure 1:**
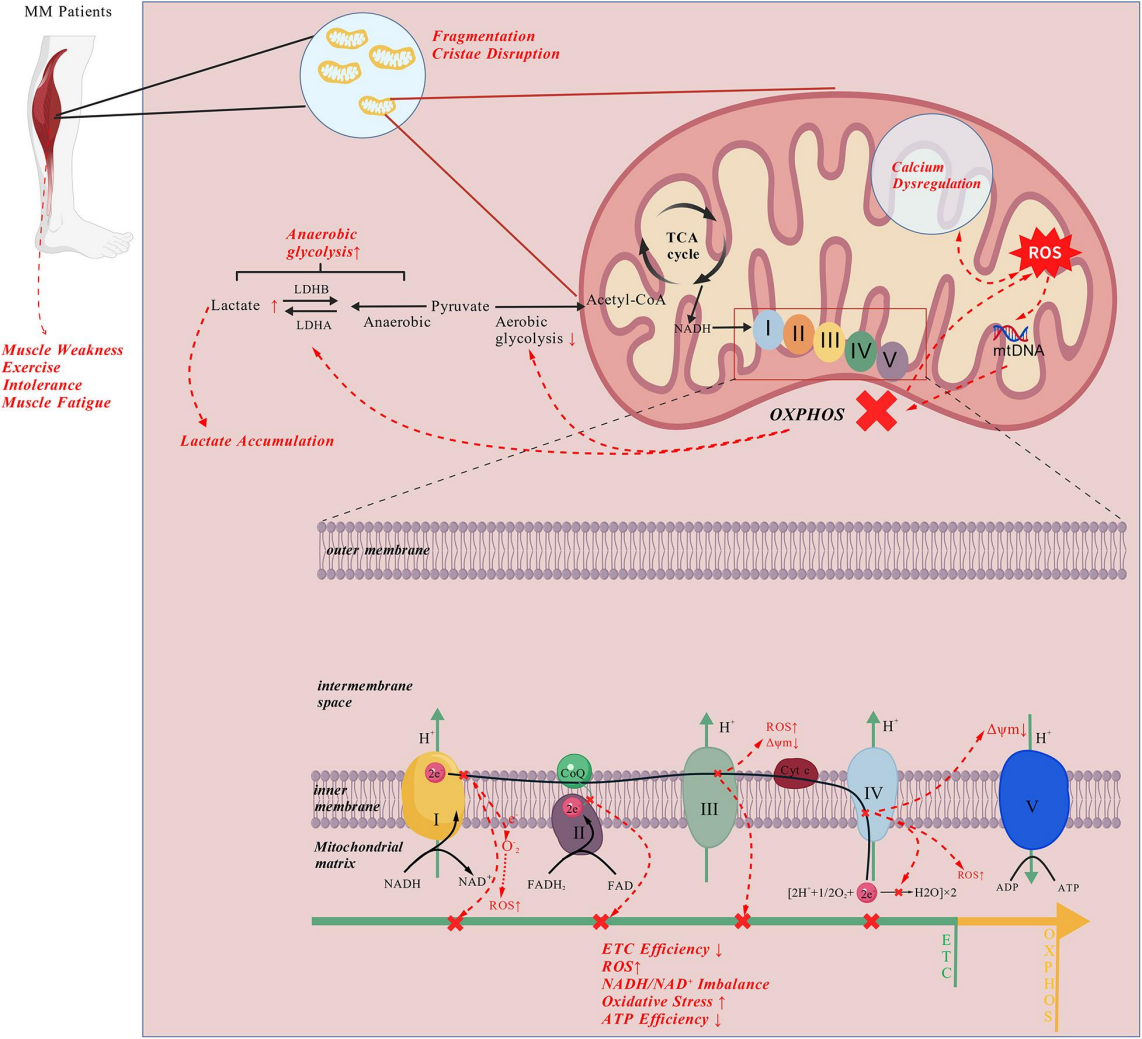
Pathological mechanisms of mitochondrial dysfunction leading to exercise intolerance in mitochondrial myopathies. The figure illustrates mitochondrial structural abnormalities (fragmentation and cristae disruption), calcium homeostasis dysregulation, genetic mutations in key complexes of the electron transport chain (ETC), and the resulting oxidative phosphorylation (OXPHOS) defects and excessive reactive oxygen species (ROS) production. Additionally, the impairment of aerobic glycolysis and the tricarboxylic acid (TCA) cycle lead to compensatory upregulation of anaerobic glycolysis, resulting in lactate accumulation. These metabolic disturbances ultimately cause insufficient energy supply, manifesting as muscle weakness and exercise intolerance, which are characteristic clinical features (Created with BioGDP.com; ID: GDP2025CWJ2W7).

Clinically, MM manifests as exercise intolerance, premature fatigue, and progressive muscle weakness (17). Impaired oxidative metabolism leads to rapid depletion of phosphocreatine and accumulation of lactate even under mild activity, reflecting a metabolic shift toward anaerobic energy production. Prolonged inactivity and ATP deficiency contribute to muscle atrophy and structural abnormalities such as ragged-red fibers (18, 19), which collectively reduce physical performance and quality of life (Figure 1) (20).

It is well established that both aerobic and resistance exercise can effectively improve the metabolic function of the skeletal muscle system (21). Aerobic exercise can effectively enhance the oxidative metabolic function of skeletal muscle. The expression levels of mitochondrial biogenesis-related protein factors significantly increase with the intensity of aerobic exercise (22). Moderate- to low-intensity aerobic exercise supports mitochondrial antioxidant balance by reducing oxidative stress and apoptosis in skeletal muscle, thereby helping to preserve muscle mass. Although high-intensity aerobic exercise increases ROS production, it more effectively enhances mitochondrial density and the activity of ETC-related enzymes (23).

Moreover, resistance exercise can stimulate muscle growth and promote muscle hypertrophy (24). In addition to its anabolic effects, resistance exercise has been reported to enhance mitochondrial biogenesis and regulate mitochondrial network morphology, including fusion and fission dynamics (21, 25). These changes are mediated by signaling pathways involving AMP-activated protein kinase (AMPK), peroxisome proliferator-activated receptor gamma coactivator 1-alpha (PGC-1*α*), and mitochondrial dynamics proteins such as Dynamin-related protein 1 (DRP1) and Optic Atrophy 1 (OPA1). Notably, the impact of resistance exercise on mitochondrial and metabolic adaptations varies by intensity. Moderate- to low-intensity resistance exercise is more associated with favorable mitochondrial remodeling and improvements in metabolic flexibility, potentially by enhancing oxidative capacity and substrate utilization efficiency (26). In contrast, high-intensity resistance training predominantly strengthens the phosphagen system and anaerobic glycolytic pathways, improving short-duration high-power output.

The effects of exercise vary depending on the method, frequency, intensity, and duration of the exercise, leading to differences in regulatory outcomes. Acute exercise can temporarily enhance mitochondrial function, increasing ATP production and oxidative metabolism rates (27). However, it cannot increase protein accumulation levels, and high-intensity acute exercise, due to differences in adaptability, may lead to negative effects such as mitochondrial oxidative stress, calcium imbalance, OXPHOS dysfunction, and skeletal muscle fiber damage (28). Short-term exercise gradually increases mitochondrial density (29) and improves the antioxidant system (30). Long-term exercise can lead to sustained mitochondrial adaptations, including an increase in mitochondrial quantity, enhanced oxidative capacity, and improved metabolic function (31).

In patients with MM, these adaptive mechanisms are limited by the functional reserve of residual intact mitochondria. The degree of benefit from exercise depends on mutation load and phenotype-specific oxidative capacity (32, 33), which may explain the variability observed among studies (34). Studies have shown that aerobic exercise can increase citrate synthase (CS) activity in patients with MM. Resistance exercise can improve muscle mass and strength, alleviating symptoms in patients with MM However, concerns remain that excessive or improperly prescribed exercise could exacerbate oxidative damage in severely affected individuals. In particular, high-intensity or unaccustomed acute exercise may trigger severe muscle injury and rhabdomyolysis (RML) due to impaired ATP resynthesis and defective mitochondrial buffering of metabolic stress (35, 36). Therefore, the optimal exercise regimen for MM patients remains to be defined.

This study examines the effects of different exercise prescription components (type, intensity, duration, cycle, and frequency) on MM and explores how exercise influences mitochondrial biogenesis, oxidative stress regulation, and muscle function across distinct MM phenotypes. This study hypothesizes that personalized and progressively structured exercise programs tailored to disease phenotype may contribute to improved functional outcomes and quality of life. This study aims to summarize the preliminary available data that may support future exploration of exercise-based therapeutic strategies for MM.

## 2 Methods

### 2.1 Protocol and registration

This systematic review followed the Preferred Reporting Items for Systematic Reviews and Meta-Analyses (PRISMA) 2020 guidelines (37) and was registered in PROSPERO (Registration No. CRD420251145502).

### 2.2 Search strategy

We searched the PubMed, Web of Science, Embase, and Scopus databases from January 1, 1990, to September 2025. A search strategy was developed by combining keywords and Medical Subject Headings (MeSH), incorporating disease-related terms (“mitochondrial myopathy,” “mitochondrial disease,” “metabolic myopathy”) and exercise-related terms (“exercise,” “exercise therapy,” “aerobic,” “resistance,” “physical activity”). To better capture interventional studies, we further included terms such as (“trial,” “intervention,” “randomized,” “exercise program,” and “exercise therapy”).

The search was restricted to human studies and limited to articles published in English. Only studies that implemented a complete training program were included, while single-session and acute exercise studies were excluded. The analysis was limited to adult MM patients (aged ≥18 years). Reference lists of all eligible articles and relevant reviews were manually screened to identify additional studies not captured by the database search. The detailed search strategy is provided in Supplementary Material (Supplementary File S1).

### 2.3 Eligibility criteria

The inclusion and exclusion criteria are summarized in Table 1. Eligible studies included adult patients (≥18 years) with clinically or genetically confirmed MM. Only interventional studies that implemented a complete exercise training program, including aerobic, resistance, or combined training, and clearly reported training duration, frequency, and intensity, were included. Studies that involved pharmacological or nutritional therapy alone, or evaluated only a single exercise session or acute bout, were excluded. To be eligible, studies had to report at least one exercise-related outcome, such as aerobic capacity, muscle strength, enzymatic or mitochondrial activity, or biochemical indices. Acceptable study designs included randomized controlled trials, non-randomized controlled trials, pre–post studies, and case series involving two or more participants, while single case reports, reviews, conference abstracts, and editorials were excluded. Only peer-reviewed full-text articles published in English were considered, and studies without accessible full texts were excluded.

**Table 1:** Inclusion and exclusion criteria for study selection.

Domain	Inclusion criteria	Exclusion criteria
Population	Adult patients (≥18 years) with clinically or molecularly confirmed	Animal or cell studies; participants without a confirmed MM diagnosis
Intervention	Exercise interventions that implemented a complete training program, including aerobic, resistance, or combined training	Non-exercise–based interventions (e.g., pharmacological treatments, nutritional supplements)
Outcomes	Reported exercise-related outcomes, including exercise capacity, muscle function, mitochondrial function, or clinical features	Studies without complete outcome data or with missing primary endpoints
Study design	Randomized controlled trials (RCTs), non-randomized controlled trials, pre–post studies, and case series (≥2 cases)	Single case reports (*n* = 1), reviews, conference abstracts, editorials, book chapters
Publication	Peer-reviewed full-text articles published in English	Non-English publications or studies without accessible full text

### 2.4 Study selection and data extraction

**Figure 2:**
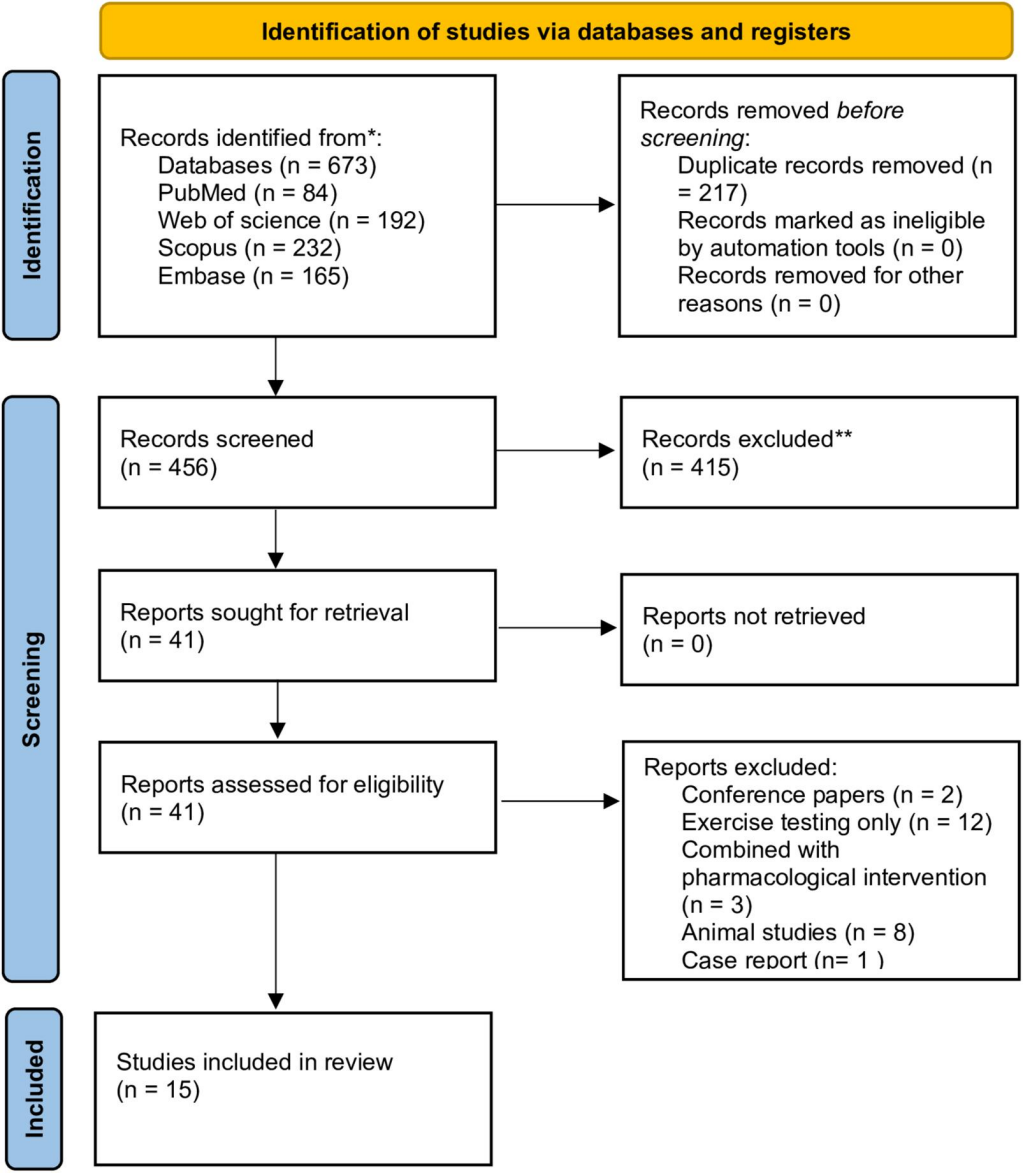
PRISMA 2020 flow diagram of study selection.

Two reviewers (Q.W. and Y.D.) independently screened the titles and abstracts to identify and exclude studies not meeting the inclusion criteria. The full texts of potentially eligible studies were subsequently assessed in detail. Any disagreements were resolved through discussion with a third reviewer (W.L.). The study selection process is presented in the PRISMA flow diagram (Figure 2).

Data extraction was independently performed by two reviewers (Q.W. and Y.D.) using a standardized form. Extracted data included study characteristics (author, year, study design), patient characteristics (sample size, sex, age, MM phenotype), intervention details (exercise type, intensity, duration, frequency), and primary outcome measures (exercise capacity, muscle function, mitochondrial function indicators). Any discrepancies or missing data were addressed through discussion or, when possible, by contacting the original authors.

### 2.5 Quality and risk of bias assessment

**Figure 3:**
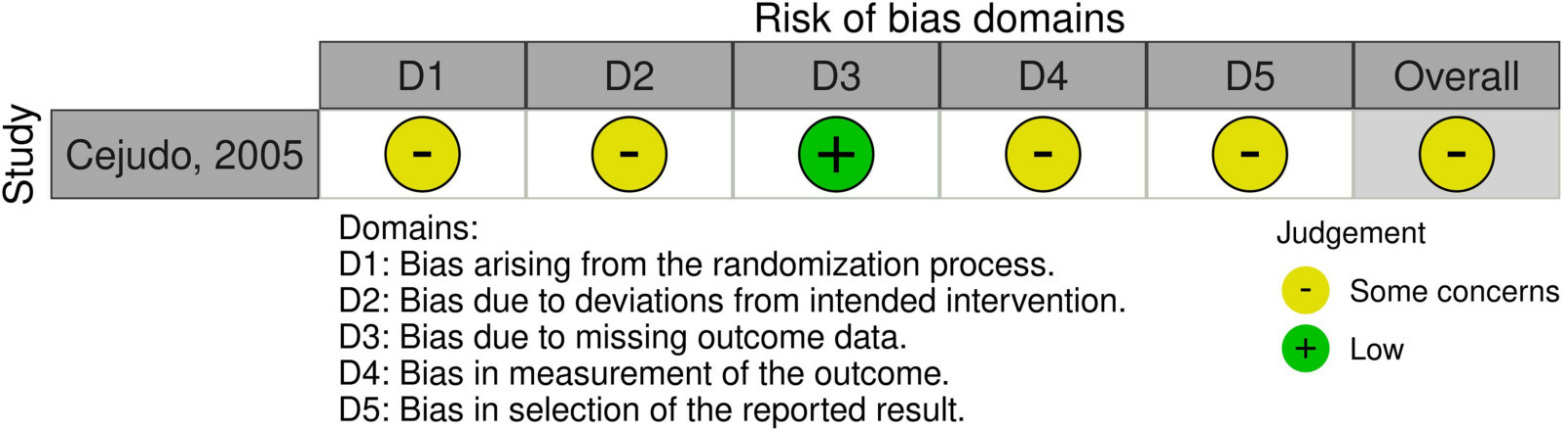
Risk of bias assessment of the included randomized controlled trial using the RoB 2 tool.

**Figure 4:**
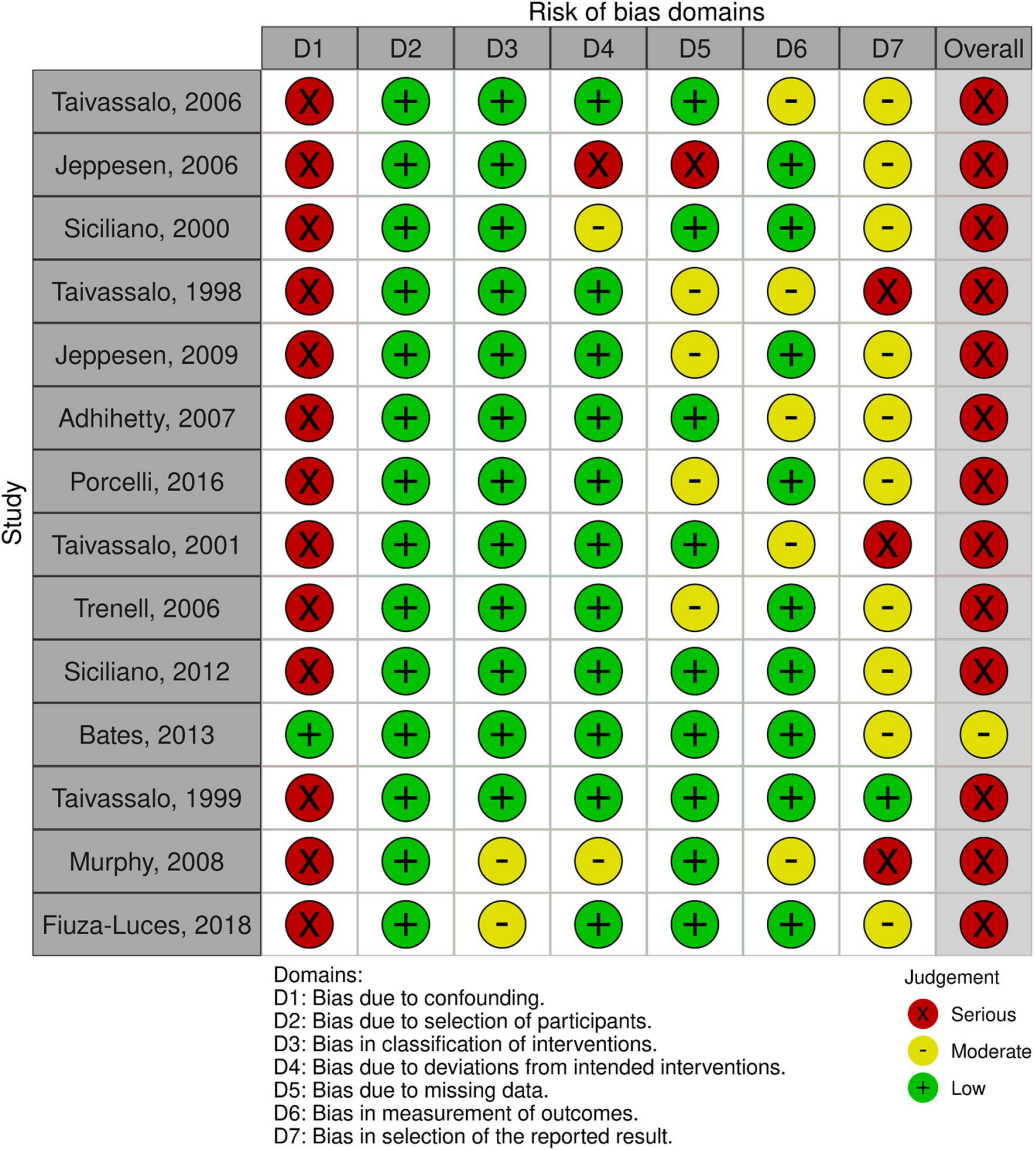
Risk of bias assessment of the included non-randomized studies using the ROBINS-I V2 tool.

Two reviewers (Q.W. and Y.D.) independently assessed the risk of bias and methodological quality. For randomized controlled trials (RCTs), the RoB 2 tool was applied (38), while for non-RCTs, the ROBINS-I V2 tool was used (39, 40). Overall methodological quality was appraised using the MMAT tool (41). Any discrepancies between the two reviewers were resolved through discussion with a third reviewer (W.L.). The results of risk of bias and quality assessment are presented in both graphical and tabular formats for clarity. The RoB 2 and ROBINS-I V2 outcomes were summarized in risk of bias figures (Figures 3, 4), while the MMAT quality appraisal results were presented in two tables (Tables 2, 3).

**Table 2:** Methodological quality appraisal of non-randomized studies (MMAT).

Study	D.1	D.2	D.3	D.4	D.5
Taivassalo et al. (45)	Can’t tell	Yes	Yes	No	Yes
Jeppesen et al. (48)	No	Yes	Yes	No	Yes
Siciliano et al. (46)	Can’t tell	Yes	Yes	No	Yes
Taivassalo et al. (42)	No	Yes	Yes	Yes	No
Jeppesen et al. (49)	No	Yes	Yes	No	Yes
Adhihetty et al. (50)	Can’t tell	Yes	Yes	No	Yes
Porcelli et al. (52)	No	Yes	Yes	No	Yes
Taivassalo et al. (44)	Yes	Yes	Yes	No	Yes
Trenell et al. (32)	Can’t tell	Yes	Yes	No	Yes
Siciliano et al. (47)	Can’t tell	Yes	Yes	No	Yes
Bates et al. (51)	Yes	Yes	Yes	Can't tell	Yes
Taivassalo et al. (43)	Can’t tell	Yes	Yes	No	Yes
Murphy et al. (33)	Yes	Yes	Yes	No	Yes
Fiuza-Luces et al. (54)	Can’t tell	Yes	Yes	No	Yes

D.1 Are the participants representative of the target population?.

D.2 Are measurements appropriate regarding both the outcome and intervention (or exposure)?.

D.3 Are there complete outcome data?.

D.4 Are the confounders accounted for in the design and analysis?.

D.5. During the study period, is the intervention administered (or exposure occurred) as intended?.

**Table 3:** Methodological quality appraisal of randomized controlled trials (MMAT).

Study	D.1	D.2	D.3	D.4	D.5
Cejudo et al., (53)	Can't tell	Yes	Yes	Can't tell	Yes

D.1 Is randomization appropriately performed?.

D.2 Are the groups comparable at baseline?.

D.3 Are there complete outcome data?.

D.4 Are outcome assessors blinded to the intervention provided?.

D.5 Did the participants adhere to the assigned intervention?.

### 2.6 Analysis and methods

Due to the substantial heterogeneity among the included studies, the findings were synthesized using a narrative approach. The heterogeneity primarily resulted from differences in outcome measures, exercise protocols, and patient characteristics across studies. In particular, the included studies assessed exercise capacity, physiological responses, and metabolic adaptations using a variety of measurement approaches and testing procedures. Training interventions also varied considerably in terms of exercise modality, intensity, frequency, and duration. Therefore, outcome indicators that were reported with relatively high frequency across the included studies were extracted and summarized in supplementary tables, presenting the baseline and post-training values reported in the original studies.

## 3 Results

### 3.1 Study selection

A total of 673 records were identified, including 84 from PubMed, 192 from Web of Science, 232 from Scopus, and 165 from Embase. After removing 217 duplicates, 456 unique records remained. Following title and abstract screening, 415 records were excluded, leaving 41 full-text articles for eligibility assessment. Of these, 26 were excluded (2 conference papers, 2 studies involving exercise testing only, 3 studies combined with pharmacological interventions, 8 animal studies, and 1 case report). 15 studies were included according to the eligibility criteria. The selection process is illustrated in the PRISMA flow diagram (Figure 2).

### 3.2 Study characteristics

This review included 15 studies, comprising 1 randomized controlled trial and 14 non-randomized studies, with sample sizes ranging from 4 to 20 participants. All subjects were patients with mitochondrial myopathy (MM), encompassing single large-scale deletions, point mutations, multiple deletions, microdeletions, and unspecified subtypes. Exercise interventions involved aerobic training, resistance training, combined modalities, and inspiratory muscle training, with durations mainly between 8 and 14 weeks, though some extended beyond 12 months.

Primary outcomes included exercise capacity (e.g., maximal oxygen uptake (VO_2_ max), maximal work rate (W max), peak oxygen uptake (Peak VO_2_), six-minute walk distance (6MWD)), muscle function [e.g., one-repetition maximum (1RM), muscle cross-sectional area], mitochondrial function [e.g., citrate synthase (CS) activity, ETC complex activity], and metabolic or inflammatory markers. Overall, most studies reported improvements in exercise capacity, muscle function, and mitochondrial metabolism, with no changes observed in creatine kinase (CK) levels or mutation burden. Detailed results are presented in Table 4.

**Table 4:** Characteristics of included studies on exercise interventions in patients with mitochondrial myopathy.

No.	Design	Sample	Exercise	Key findings (Effects)	References
1	Non-RCT	*n* = 8 (5F, 3M; 37 ± 8 y, 46 ± 11 y) large-scale mtDNA deletions	Aerobic Exercise (14wk) 70%–80% Hrmax 3–4×/wk followed by 14 wk training vs Detraining	PW ↑, Peak VO_2_ ↑, Peak A-VO_2_Δ ↑, HR ↓, CS ↑(ns), Complex I / II / IV ↑(ns), Lactate ↓, mtDNA deletion % ↔	(45)
2	Non-RCT	*n* = 20 (10F, 10M; 41 ± 12 y; 64 ± 12 kg; 170 ± 11 cm) 5 single large-scale deletions 1 microdeletion 14-point mutations	Aerobic Exercise (12wk) 65%–75% VO_2_max 50 sessions/12 wk followed by 8 wk complete detraining	VO_2_max ↑, W max ↑, CS activity ↑, Complex I activity ↑ (ns), Complex IV activity ↑(ns), mtDNA ↑, mtDNA mutation load ↔, CK activity ↔	(48)
3	Non-RCT	*n* = 12 (10F, 1M; 46.7 ± 5.6 y) 6 single large-scale deletions 2 uncharacterized 3 multiple deletions 1 point mutation	Aerobic Exercise (10 wk) near-LT workload (∼40%–50% pnPOmax) 3–4×/wk, 30–45 min	rev./min ↑(ns), Lactate concentration ↓, EP ↓ (ns), NEP ↓ (ns), HR ↓ (ns), Lactate recovery AUC ↓	(46)
4	Non-RCT	*n* = 10 (4F, 6M; 36 ± 9 y) 5 deletions 2-point mutations 3 uncharacterized mtDNA (2 suspected nuclear mutations)	Aerobic Exercise (8wk) HRR 60%–80% 3–4×/wk, 20–30 min	Aerobic capacity ↑, Heart rate at rest ↓, Heart rate after exercise ↓, Blood lactate at rest ↓, Blood lactate after exercise↓, CK activity ↔, ADP t ½ ↓	(42)
5	Non-RCT	*n* = 4 (4 M; 32 ± 4 y) 1 ND2 microdeletion 1 large-scale deletion (7,177–13,767) 2-point mutations (3243A>G, 8344A>G)	Aerobic Exercise (3–12 M) 1st phase: 3 mo, 5×/wk, moderate intensity 2nd phase: 6–12 mo, 3×/wk, 30 min, ∼70% VO_2_ max Detraining: 3–12 mo between phases	VO_2_ max ↑, W max ↑, Type I fiber CSA ↑, Capillary density ↑, CS activity ↑, CK activity ↔, mtDNA/nDNA ↑, mutation load ↔	(49)
6	Non-RCT	*n* = 8 (5F, 3M; 37.6 ± 3.2 y) 4 large-scale deletions 2 microdeletions 2-point mutations	Aerobic Exercise (14wk) MHR 70%–80% 3–4×/wk	Cytochrome c ↑, mtHSP70 ↑, Bax ↑, MnSOD ↑, OGG-1 ↓, Aconitase enzyme activity ↓	(50)
7	Non-RCT	*n* = 6 (4M, 2F; 51 ± 16 y; 69.1 ± 18.1 kg; 171 ± 7 cm) Genotype not specified	Aerobic Exercise (12wk) MHR 65%–70% 4×/wk, 30–45 min	Peak Work Rate ↑, Peak VO_2_ ↑, [deoxy (Hb + Mb)] peak ↑, V˙O2 kinetics ↓, Δ [deoxy (Hb + Mb)] kinetics ↓(ns)	(52)
8	Non-RCT	*n* = 10 (6F, 4M; 39.3 ± 9.5 y) 2 single large-scale deletions 2 tRNA mutations 1 ND4 mutation 2 Cyt b mutations 1 COX I mutation 1 COX III microdeletion 1 uncharacterized	Aerobic Exercise (14 wk) MHR 70–80% 3–4×/wk, 30 → 40 min	W ↑, Peak VO_2_ ↑, systemic a–vO_2_ diff ↑, ΔQ/ΔVO_2_ ↓, PCr resynthesis ↑, CS ↑, SDH ↑, COX ↑ (in COX-deficient)	(44)
9	Non-RCT	*n* = 10 (3M, 7F; 42 ± 14 y) 5-point mutation (m.3243A>G) 5 ragged-red fibers	Aerobic Exercise (12 wk) MHR 70–80% 3×/wk, 30 min	Total muscle volume ↑, Muscle maximum area ↑, OUES ↑, Peak Watts ↑, 6-min walk distance ↑, V ↑ (PCr recovery rate), QMAX ↑	(32)
10	Non-RCT	*n* = 7 (1M, 6F; 44.9 ± 12.1 y) 4 single large-scale deletions 3 multiple deletions	Aerobic Exercise (10wk) 40% pnPOmax, 30 → 45 min	Lactate ↓ (40% pnPOmax), Lipoperoxides ↓ (rest, exercise trend)	(47)
11	Non-RCT	n = 10 (6M, 4F; 42.4 ± 10.5 y) 10 m.3243A > G	Aerobic exercise (12wk) Anaerobic threshold intensity 3×/wk, 30–45 min	PPO ↑, Peak VO_2_ ↑, Peak A–V¯O_2_ Δ ↑(ns) LVMI ↔, PCr/ATP ↔	(51)
12	Non-RCT	*n* = 14 (4M, 10F; 36.4 ± 9.8 y) 9 single large-scale deletions 2-point mutations 3 unclassified	Aerobic Exercise (8 wk) HRR 70%–85% 3–4×/wk, 20–30 min	METs ↑, HR ↓, Lactate ↓, ADP t½ ↓, CK ↔	(43)
13	Non-RCT	*n* = 8 (6M; 39 ± 9 y) large-scale mtDNA deletions	Resistance training (12wk) 80%–85% (1RM) 3×/wk, 3 sets × 6–8 reps	1RM ↑, Peak A-VO_2_ Δ ↑, Central nuclei fibers ↑, Neonatal Myosin ↑, NCAM ↑, COX-deficient fibers ↓, mtDNA mutation ↓(ns), CK ↔	(33)
14	Non-RCT	*n* = 12 (8M, 4F; 38 ± 11 y) 4 single large-scale deletions 5 multiple deletions 3-point mutations	Aerobic exercise (8wk) Intensity: 65% → 75% → 85% → 90 → 100% PPO Resistance training (8wk) Intensity: RPE 6–7/10 3×/wk, 60–90 min Inspiratory muscle training (8wk) Threshold device at 40% PI max 2×/day × 30 breaths	PPO ↑, Peak VO_2_ ↑, PImax ↑, 6MWD ↑, lean body mass ↑, Fat mass ↓, Inflammatory factors ↔, Metabolic factors ↔, CK activity ↔	(54)
15	RCT	n = 18 (10M, 8F; 44 ± 11 y) Genotype not specified	Aerobic Exercise + Resistance training (12wk) 70% max workload / 50% 1RM 3×/wk, 1h	VO_2_ max ↑, 1RM ↑, O_2_ pulse ↑, VEmax ↑, Anaerobic Threshold ↑, MIP ↑, MEP ↑	(53)

VO_2_ max, Maximal Oxygen Uptake; Peak VO_2_, Peak Oxygen Consumption; PW, Peak Work; A-VO_2_ Δ, Arteriovenous Oxygen Difference; HR, Heart Rate; CS activity, Citrate Synthase Activity; C-I activity, Complex I Activity; C-II activity, Complex II Activity; C-IV activity, Complex IV Activity; mtDNA, Mitochondrial DNA; W max, Maximum Work; CK activity, Creatine Kinase Activity; HRR, Heart Rate Reserve; ADP t ½, Half-life of ADP; MHR, Maximum Heart Rate; mtHSP70, Mitochondrial Heat Shock Protein 70; MnSOD, Manganese Superoxide Dismutase; OGG-1, 8-Oxoguanine DNA Glycosylase-1; Hb + Mb, Hemoglobin+Myoglobin; PCr recovery rate, Phosphocreatine Recovery Rate; QMAX, Maximum Cardiac Output; pnPO max, Peak Neuromuscular Power Output; LPO, Lipid Peroxidation Levels; AT, Anaerobic Threshold; 1RM, One Repetition Maximum; NCAM, Neural Cell Adhesion Molecule; COX-deficient fibers: Cytochrome c Oxidase-Deficient Fibers; PPO, Peak Power Output; 6MWD, Six-Minute Walk Distance; PI max, Maximal Inspiratory Pressure.

Among the included studies, several outcome domains were reported repeatedly across different interventions. Therefore, outcomes that appeared with relatively high frequency in the included studies were extracted and organized in Supplementary (Supplementary Tables S1–S4). These tables summarize the baseline and post-training values reported in the original studies for the outcomes most frequently assessed in the literature.

### 3.3 Risk of bias and quality appraisal

Of the 15 studies included in this review, one randomized controlled trial was assessed using the RoB 2 tool (Figure 3), which indicated a moderate risk of bias. The remaining 14 non-randomized studies were evaluated with the ROBINS-I V2 tool (Figure 4); all but one study showed a serious risk of bias, primarily due to insufficient control of confounding factors. Quality appraisal using the MMAT demonstrated that most studies performed well in addressing research questions and outcome measures (Tables 2, 3), but limitations were evident in sample size, control groups, and randomization. Given that mitochondrial myopathy is a rare disease, the included studies generally had small sample sizes, and patients exhibited considerable genetic and phenotypic heterogeneity, which increased the likelihood of potential confounding and restricted control of bias.

### 3.4 Effects of aerobic exercise

Among the included studies (Table 4), several demonstrated that (32, 42–52), in terms of mitochondrial function, aerobic exercise increased CS, succinate dehydrogenase (SDH), and cytochrome c oxidase (COX) activities in MM patients and enhanced the activities of mitochondrial complexes I, II, and IV (44, 45, 48). In energy metabolism, aerobic exercise improved phosphocreatine (PCr) recovery rate and maximum ATP synthesis rate (Q max), while shortening half-time of adenosine diphosphate recovery (ADP t½) (32, 42–44). With respect to oxidative stress, aerobic exercise elevated manganese superoxide dismutase (MnSOD) expression levels and reduced lipid peroxidation, whereas decreases were observed in 8-oxoguanine glycosylase-1 (OGG1) and histone acetyltransferase (HAT) activity (50). In muscle structure, aerobic exercise increased muscle fiber cross-sectional area and total volume, and enhanced capillary density (32). Regarding exercise capacity and cardiopulmonary function, VO_2_ max, peak power, and 6MWD were significantly improved; oxygen uptake efficiency slope (OUES) and arteriovenous oxygen difference (A-VO_2_ Δ) increased (32, 42, 44, 46–49, 51, 52); and both resting and exercise heart rates decreased. Notably, most studies did not observe increases in CK levels or mtDNA mutation burden.

Across these studies, aerobic training programs typically lasted 8–14 weeks, conducted 3–5 sessions per week, each lasting 30–60 min at 60%–80% of VO_2_ max or HRmax. A few long-term interventions extended beyond 12 months and maintained improvements in aerobic capacity and mitochondrial function (32, 42–52). Short-term programs of 8–12 weeks were sufficient to enhance aerobic capacity and metabolic function, although some studies noted that benefits declined after detraining periods.

### 3.5 Effects of resistance exercise

Among the included studies (Table 4), a limited number investigated the effects of resistance exercise in MM patients (33). These studies reported significant improvements in muscle strength, enhanced satellite cell (SC) activity, increased proportions of centrally nucleated and newly formed myosin heavy chain (MyHC)-positive fibers, and a marked reduction in COX-deficient fibers, while overall mitochondrial DNA mutation levels and CK concentrations remained unchanged. Additionally, combined aerobic and resistance exercise interventions were shown to improve mitochondrial enzyme activity and cardiovascular function, while concurrently increasing muscle mass and strength.

Resistance training protocols were generally 8–12 weeks in duration, performed three times per week, and typically consisted of three sets of 6–8 repetitions at 70%–85% of 1RM (33, 53, 54). These moderate-to-high-intensity loads produced strength and hypertrophic adaptations without raising CK levels or mtDNA mutation burden. Longer interventions and progressive loading appeared to support more sustained muscle and metabolic benefits over time.

### 3.6 Phenotype-specific responses

Among the included studies, only a few specifically examined differences across MM phenotypes or focused on a single phenotype, and these studies generally had small sample sizes (Table 5).

**Table 5:** Phenotype-specific responses to exercise interventions in mitochondrial myopathy.

Treatment	Exercise effects	References
Aerobic Exercise (14wk) 70%–80% HRmax 3–4×/wk	Single large-scale deletions (*n* = 8): PW ↑; Peak VO_2_ ↑; Peak A-VO_2_Δ ↑; HR ↓CS ↑(ns); Complex I / II / IV ↑(ns) Lactate ↓ mtDNA deletion % ↔	(45)
Resistance training (12wk) 80%–85% (1RM) 3×/wk, 3 sets × 6–8 reps	Single large-scale deletions (*n* = 8): 1RM ↑, Peak A-VO_2_ Δ ↑, Central nuclei fibers ↑, Neonatal Myosin ↑, NCAM ↑, COX-deficient fibers ↓	(33)
Aerobic exercise (12wk) Anaerobic threshold intensity 3×/wk, 30–45 min	Point mutation 3243A>G (n = 10): PPO ↑, Peak VO_2_ ↑, Peak A-V¯O_2_ Δ ↑ LVMI ↔, PCr/ATP ↔	(51)
Aerobic Exercise (12wk) 65%–75% VO2max 50 sessions/12 wk followed by 8 wk complete detraining	Single large-scale deletions (*n* = 5): Aerobic capacity ↑, W max ↑, CS ↑, mtDNA/nDNA ↑, mutation load ↔ or ↓ after detraining, no adverse morphological change. Effect: good. Point mutation 3243A>G (*n* = 13): Aerobic capacity ↑, W max ↑, CS ↑, mtDNA/nDNA ↑, mutation load ↔ or ↓. Effect: good. Point mutation 8344A>G (*n* = 1): Aerobic capacity ↑, W max ↑, mtDNA/nDNA ↑, mutation load ↑ (∼4%) after detraining, phenotype more complex. Effect: uncertain.	(48)
Aerobic Exercise (8 wk) HRR 60%–80% 3–4×/wk, 20–30 min	Large-scale deletions (*n* = 5): Aerobic capacity ↑, exercise duration ↑, HR_rest ↓, HR_iso ↓, lactate ↓ (variable; one case limited), ADP t½ ↓. Effect: moderate. Point mutations (*n* = 2): Aerobic capacity ↑, HR ↓, exercise lactate ↓, resting lactate ↑, ADP t½ ↓. Effect: moderate. Uncharacterized mtDNA (*n* = 3; incl. 2 suspected nuclear): Aerobic capacity ↑ (∼50%), HR ↓, lactate ↓, ADP t½ ↓ (∼80%). Effect: best.	(42)
Aerobic Exercise (3–12 M) 1st phase: 3 mo, 5×/wk, moderate intensity 2nd phase: 6–12 mo, 3×/wk, 30 min, ∼70% VO_2_ max Detraining: 3–12 mo between phases	ND2 microdeletion (*n* = 1): Aerobic capacity ↑, W max ↑, mtDNA/nDNA ↑ (3M & 12M), mutation load ↔, adherence lowest. Effect: best. Large-scale deletion 7,177–13,767 (*n* = 1): Aerobic capacity ↑, W max ↑, mtDNA/nDNA ↑ (3M only), mutation load ↔, no long-term gain. Effect: moderate. Point mutation 3243A > G (*n* = 1): Aerobic capacity ↑, W max ↑, mtDNA/nDNA ↑ (3M only), mutation load ↔, no further change. Effect: limited. Point mutation 8344A > G (*n* = 1): Aerobic capacity ↑, W max ↑, mtDNA/nDNA ↑ (3M & 12M), mutation load ↔. Effect: good.	(49)
Aerobic Exercise (14 wk) MHR 70%–80% 3–4×/wk, 30 → 40 min	Large-scale deletions (*n* = 2): W ↑, Peak VO_2_ ↑, CS ↑, SDH ↑, COX ↔/↑, mtDNA mutation load ↑ (small), effect overall positive. Effect: good–moderate. Microdeletion COX III (*n* = 1): W ↑, Peak VO_2_ ↑, COX ↑ (+77%), CS ↑, SDH ↑, mutation load ↑ (small). Effect: good. Point mutations (*n* = 6): tRNA mutations (n = 2): W ↑, Peak VO_2_ ↑, COX ↑ (+30%), mutation load ↔/↑, effect positive. Effect: good. ND4 mutation (*n* = 1, Complex I): W ↑, Peak VO_2_ ↑, Complex I ↑ (+36%), but relative to CS ↓, mutation load ↑. Effect: moderate. Cyt b mutations (*n* = 2, Complex III): W ↑ (variable), Peak VO_2_ ↓/↔, Complex III ↓ (−20%), mutation load ↑ (up to +50%), weakest adaptation. Effect: poor. COX I mutation (*n* = 1): W ↑, Peak VO_2_ ↑, COX ↑ (+16%), mutation load ↑. Effect: moderate.	(44)

VO_2_ max, Maximal Oxygen Consumption; Peak VO_2_, Peak Oxygen Uptake; PW, Peak Work; A-VO_2_Δ, Arteriovenous Oxygen Difference; HR, Heart Rate; HR_rest, Resting Heart Rate; HR_iso, Isotime Heart Rate; HRR, Heart Rate Reserve; CS, Citrate Synthase Activity; SDH, Succinate Dehydrogenase Activity; COX, Cytochrome c Oxidase Activity; Complex I, NADH Dehydrogenase Activity; Complex II, Succinate Dehydrogenase Activity; Complex III, Cytochrome bc1 Complex Activity; Complex IV, Cytochrome c Oxidase Activity; 1RM, One Repetition Maximum; PPO, Peak Power Output; W max (W), Maximum Workload; LVMI, Left Ventricular Mass Index; PCr/ATP, Phosphocreatine to Adenosine Triphosphate Ratio; PCr, Phosphocreatine; ADP t½, Half-life of ADP Recovery; mtDNA, Mitochondrial DNA; nDNA, Nuclear DNA; NCAM, Neural Cell Adhesion Molecule; Cyt b, Cytochrome b; ND2, NADH Dehydrogenase Subunit 2; ND4, NADH Dehydrogenase Subunit 4.

Mutation subgroups were classified according to the molecular type and functional impact of the mutations reported in the included studies (33, 42, 44, 45, 48, 49, 51), comprising large-scale deletions (≥5 kb, partially retaining normal mtDNA and residual OXPHOS activity), microdeletions (<1 kb within coding regions such as ND2 or COX III), and point mutations (single-base substitutions such as m.3243A>G, m.8344A>G, ND4, COX I, or Cyt b) that disrupt specific ETC subunits or tRNA genes. This classification reflects their distinct effects on oxidative phosphorylation and phenotype, where large-scale deletions generally preserve compensatory mitochondrial capacity, tRNA mutations exhibit moderate adaptability, and mutations directly affecting ETC complexes (e.g., Cyt b or COX I) often result in poor or adverse exercise responses.

For MM patients with large-scale deletions (33, 42, 44, 45, 48, 49), studies indicated that both aerobic and resistance exercise improved exercise capacity, including Peak VO_2_, W max, and ADP recovery kinetics, enhanced mitochondrial enzyme activities such as CS, SDH, and COX, and reduced blood lactate levels. In addition, resistance exercise increased muscle strength, the proportion of centrally nucleated fibers, and newly formed myosin-positive fibers, while reducing the proportion of COX-deficient fibers, without increases in CK levels or mutation burden.

For MM patients with point mutations (m.3243A>G, m.8344A>G, ND4, Cyt b, COX I), considerable heterogeneity was observed among subtypes (42, 44, 48, 49, 51). In patients with the m.3243A>G mutation, aerobic exercise improved exercise capacity [aerobic capacity, W max, peak power output (PPO), Peak VO_2_] and oxygen utilization (peak A-V¯O_2_ *Δ*), while increasing both mtDNA and nDNA levels without elevating mtDNA mutation load. In patients with the m.8344A>G mutation, regular aerobic training enhanced aerobic capacity and maximal power, and also increased mtDNA and nDNA levels; however, a rise in mutation burden was observed during the detraining phase. Another study reported that in MM patients with tRNA mutations, aerobic exercise improved exercise capacity (W, Peak VO_2_) and mitochondrial enzyme activity (COX). For COX I and ND4 mutations, only exercise capacity improved, but mutation burden also increased. In contrast, in patients with Cyt b mutations, aerobic exercise exerted negative effects on exercise capacity, mitochondrial function, and mutation burden.

For MM patients with microdeletions (Microdeletion COX III, ND2 microdeletion), limited evidence indicated that aerobic exercise improved exercise capacity and mitochondrial function (44, 49). In patients with ND2 microdeletion, mutation burden increased, whereas in those with Microdeletion COX III, only a slight increase in mutation burden was observed. For patients with unspecified mtDNA mutations (including two cases suspected to be nuclear gene–related), significant improvements were reported in exercise performance and metabolic capacity [reduced heart rate (HR), lactate, and ADP t½]. Overall, patients with large-scale deletions and the m.3243A>G point mutation exhibited favorable training responses, whereas those with other point mutations, microdeletions, or rare genotypes demonstrated more variable or even adverse adaptations.

## 4 Discussion

### 4.1 Types of exercise—aerobic exercise

Aerobic exercise regulates mitochondrial function mainly through activation of peroxisome proliferator-activated receptor gamma coactivator 1-alpha (PGC-1*α*), the principal regulator of mitochondrial biogenesis in skeletal muscle (55). In healthy individuals, exercise-induced PGC-1*α* activates nuclear respiratory factors and mitochondrial transcription factor A (TFAM), promoting mtDNA replication, synthesis of respiratory-chain complexes, and enhanced OXPHOS efficiency (56). It also facilitates fatty-acid oxidation and the tricarboxylic-acid (TCA) cycle, providing more substrates for OXPHOS (57). Furthermore, exercise increases antioxidant enzyme expression such as superoxide dismutase (SOD) and catalase (CAT) (58), which mitigates ROS induced mitochondrial injury and helps maintain redox balance (30). Aerobic training also improves oxygen delivery through elevated expression of vascular endothelial growth factor (59) and hypoxia inducible factor 1 alpha (60), promoting skeletal muscle capillarization and enhancing endurance (61).

**Figure 5:**
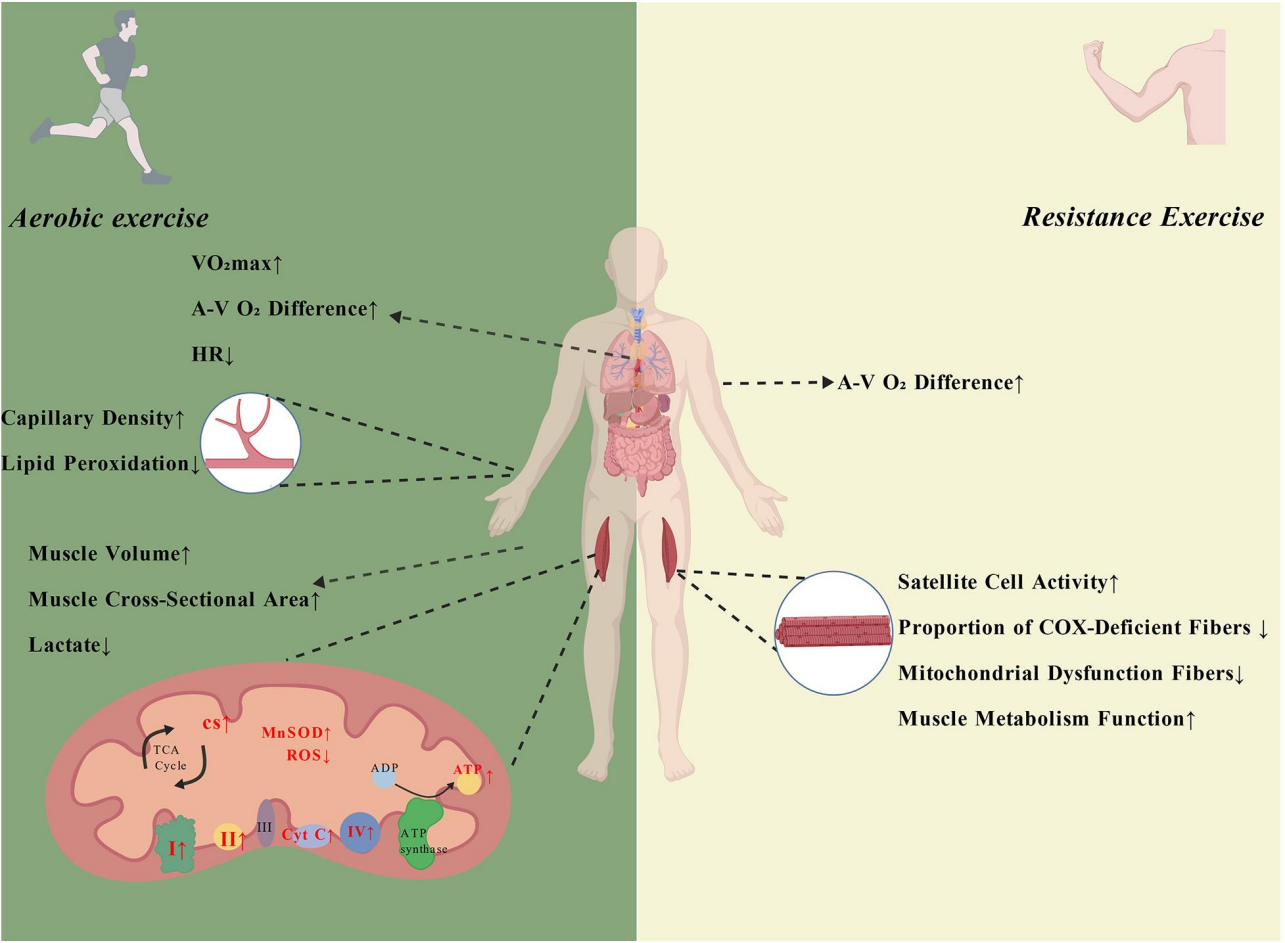
The regulatory effects of aerobic and resistance exercise on MM patients. Aerobic exercise can enhance the maximum oxygen uptake, arteriovenous oxygen difference, capillary density, muscle mass, and mitochondrial oxidative metabolism in MM patients, while lowering heart rate, lipid peroxidation, and lactate levels. Resistance exercise can improve satellite cell activity, muscle repair capacity, muscle fiber regeneration, and metabolic function, while reducing the proportion of COX-deficient and dysfunctional mitochondrial fibers (Created with BioGDP.com; ID: GDP20255WEH7V).

Current evidence indicates that aerobic exercise exerts beneficial effects on MM patients by improving mitochondrial function, energy metabolism and recovery, oxidative stress, muscle structure and circulation, as well as exercise capacity and cardiopulmonary parameters (Table 4 and Figure 5).

In terms of mitochondrial function, aerobic exercise improves the impaired oxidative metabolism in MM patients by enhancing mitochondrial enzyme activity. Previous studies have demonstrated that aerobic exercise increases the activity of CS, SDH, and COX (44, 45, 48, 49), which are classical markers of mitochondrial content and oxidative phosphorylation capacity. These findings indicate partial compensation for impaired ETC function, thereby enhancing the efficiency of ATP production. This improvement does not appear to alter the underlying mtDNA mutation load, suggesting that the benefits of aerobic exercise mainly arise from enhanced function of intact mitochondria rather than correction of the mutations themselves. Such improvement is consistent with findings in MM mouse models, where aerobic exercise upregulated key regulators of mitochondrial biogenesis, including PGC-1*α* and TFAM (62). It is noteworthy that the extent of improvement in mitochondrial enzyme activity varies across studies. This variability is partly due to the small sample sizes of existing investigations and partly to the pronounced heterogeneity of MM phenotypes. Overall, the benefits of aerobic exercise are limited by the functional reserve beyond the mutation load in MM patients, and the magnitude of improvement is largely determined by the underlying genotype (48, 49).

MM patients experience exercise intolerance and delayed post-exercise recovery as a result of defective oxidative phosphorylation, which limits ATP production (17). Existing studies have demonstrated that aerobic exercise enhances PCr resynthesis and reduces ADP t½ in MM patients (32, 42–44), These effects primarily reflect an accelerated rate of ATP regeneration driven by improved mitochondrial metabolism, which is particularly critical in individuals with limited ATP availability. Such adaptations contribute to reduced exercise-induced fatigue and greater exercise tolerance, and notably, they occur without an increase in CK levels, indicating that muscle damage is not exacerbated. Since these improvements stem from enhanced mitochondrial function, they are likewise constrained by and dependent on the functional reserve of residual intact mitochondria, with the extent of benefit influenced by the patient's genotype.

MM patients typically exhibit elevated oxidative stress due to ETC defects, resulting in excessive ROS production that damages mtDNA. This damage exacerbates energy failure and accelerates muscle fiber degeneration, thereby reinforcing the vicious cycle of mitochondrial dysfunction in MM (63). Evidence indicates that aerobic exercise elevates MnSOD levels (50) and decreases systemic lipid peroxidation markers (47, 52), suggesting a compensatory upregulation of antioxidant defenses and mitigation of oxidative damage in skeletal muscle. The reduction of ROS may contribute to the improved functional tolerance observed after exercise. However, decreased levels of OGG1 and reduced HAT activity (50) indicate that, despite enhanced antioxidant enzyme activity, overall ROS levels are not fully suppressed, and aerobic exercise alone cannot completely reverse the pathological state of MM. The upregulation of antioxidant responses can partially counteract oxidative stress–induced cellular damage. while aerobic exercise alleviates oxidative stress to some extent, persistent ROS may necessitate adjunctive antioxidant or pharmacological interventions.

Beyond mitochondrial bioenergetics and antioxidant adaptations, exercise training may also exert broader metabolic benefits in patients with mitochondrial myopathy. By enhancing mitochondrial oxidative capacity and activating insulin-independent pathways (e.g., AMPK), exercise facilitates improved glucose handling and reduces the accumulation of intracellular lipid intermediates, thereby lowering the risk of secondary metabolic disorders such as type 2 diabetes (64). This translational benefit is particularly relevant for the long-term management of patients with mitochondrial dysfunction, who are predisposed to systemic metabolic decline (65).

In terms of skeletal muscle, MM patients exhibit muscle atrophy and muscle weakness. Both muscle volume and muscle cross-sectional area are reduced compared to healthy individuals (32). There are abnormal changes in the ratio of type I endurance muscle fibers to type II strength muscle fibers (66). These structural constraints limit oxygen delivery and substrate utilization in MM patients, thereby exacerbating exercise intolerance. Aerobic exercise has been shown to increase muscle fiber cross-sectional area and overall muscle volume, partially reversing disuse atrophy (32), In addition, it enhances capillary density, improves microvascular oxygen delivery, and facilitates substrate exchange (49).

In addition to defects in oxidative phosphorylation, exercise intolerance in MM patients is influenced by impaired peripheral oxygen utilization (67, 68). Current evidence indicates that aerobic exercise improves VO_2_ max, W max, and 6MWD in MM patients. Submaximal indices such as OUES are also enhanced. In addition, aerobic exercise lowers resting and exercise heart rate (42–45, 48, 49, 54). At the same time, the widened A-VO_2_ *Δ*, together with increases in muscle fiber cross-sectional area, overall muscle volume, and capillary density, reflects more effective oxygen extraction and utilization at the muscular level. These adaptations are not only structural but also indicate enhanced peripheral oxygen utilization efficiency. Improvements in PCr resynthesis and accelerated oxygen kinetics further support the notion that peripheral functional adaptations drive the systemic benefits observed. Moreover, CK levels did not increase. The gains in VO_2_ max, W max, 6MWD, and cardiopulmonary efficiency translate directly into reduced fatigue and greater independence in the daily lives of MM patients.

Overall, aerobic exercise exerts beneficial effects across multiple domains in MM patients. These adaptations collectively translate into improved exercise tolerance and reduced daily fatigue, without increasing CK levels or mtDNA mutation load. However, the magnitude of these benefits is shaped by the heterogeneity of genetic mutations in MM, underscoring the need for individualized exercise prescriptions tailored to specific phenotypes.

### 4.2 Types of exercise—resistance exercise

When skeletal muscles undergo mechanical overload during exercise, the cross-sectional area of muscle fibers can increase, a process known as “hypertrophy” (69). This is primarily attributed to resistance exercise stimulating muscle protein synthesis, mainly through the activation of the mechanistic target of rapamycin (mTOR) signaling pathway, which enhances translational regulation and protein synthesis (70). mTOR exists in mTORC1 and mTORC2 complexes, with mTORC1 serving as the primary regulatory factor for translation initiation. It coordinates with two downstream substrates, ribosomal protein S6 and p70 kinase, as well as eukaryotic initiation factor 4E-binding protein 1, to promote protein synthesis (71).

Resistance exercise can also stimulate intracellular signaling in muscle cells, promoting mitochondrial biogenesis, although its effects are less pronounced compared to aerobic endurance exercise (72–74). Resistance exercise can effectively and significantly enhance mitochondrial respiratory capacity by improving the coupling efficiency of mitochondrial complex I and complex II (21). Resistance exercise can also increase the levels of the mitochondrial fusion marker, OPA1. Additionally, it can elevate the levels of mitochondrial fission markers, including DRP1 and Mitochondrial fission 1 protein, thereby maintaining mitochondrial structure (75, 76). Studies have also shown that resistance exercise can enhance the activity of mitochondrial ETC complexes and antioxidant enzymes, improving the efficiency of the ETC and reducing oxidative stress (77). The mitochondrial regulatory effects induced by resistance exercise are more pronounced in older adults and individuals with chronic diseases (78).

In contrast to aerobic exercise, resistance training has a more limited effect on enhancing mitochondrial enzyme activity in MM patients. Resistance exercise reduces the proportion of COX-deficient fibers (33), but this reduction occurs through the regeneration of muscle fibers enriched with normal mtDNA, thereby improving mitochondrial function rather than directly correcting the genetic defect.

Impaired peripheral oxygen utilization, rather than cardiopulmonary limitation, is a prominent feature of exercise intolerance in MM patients. From a metabolic perspective, resistance training increases the A-VO_2_ *Δ* (33), indicating improved muscular oxygen extraction and greater efficiency of oxygen use.

One of the most prominent features of MM patients is muscle weakness. Resistance training enhances muscle strength and functional reserve by increasing SC activity, as reflected by elevated numbers of centrally nucleated fibers and newly formed MyHC-positive fibers. These adaptations indicate activation of muscle regeneration and repair, leading to increased muscle mass and partially counteracting atrophy. Importantly, even under high-intensity conditions, neither CK levels nor mtDNA mutation load were aggravated (33). Thus, the primary value of resistance training in MM lies in restoring strength, promoting muscle fiber regeneration, and improving mitochondrial function, all dependent on SC-mediated expansion of genotype-normal mtDNA.

In addition to improvements in muscle strength and oxidative metabolism, resistance exercise may also exert protective effects at the neuromuscular level. Recent evidence suggests that physical activity helps preserve neuromuscular junction integrity, ensuring effective neural drive and preventing the exacerbation of muscle fiber degeneration (79).

These benefits may produce additive effects when combined with aerobic exercise. Studies incorporating both modalities (53, 54) reported improvements in mitochondrial enzyme activity and cardiopulmonary fitness, along with gains in muscle strength and mass. However, responses vary across MM phenotypes, underscoring the need for individualized exercise prescriptions based on phenotype and mutation burden.

### 4.3 Exercise duration, cycles and intensity

It is well known that a single acute exercise session can stimulate mitochondrial-related proteins, improve mitochondrial function, and enhance skeletal muscle metabolic capacity. However, these effects are not sustained (80–82). High-intensity acute exercise generates a large amount of ROS (83), which may exacerbate the pre-existing oxidative stress levels in MM patients. Acute exercise places a high demand on energy (84). MM patients have insufficient ATP production, which may exacerbate their metabolic burden and lead to fatigue. Acute and high-intensity exercise may also induce RML in patients with MM, which can be life-threatening (35, 36).

Both short-term and long-term exercise can more effectively improve mitochondrial function, increase mitochondrial quantity, and enhance muscle metabolic capacity and fatigue resistance (85–87). Especially long-term exercise, which can reduce the risk of mitochondrial aging (88).

Current evidence indicates that short-term interventions of 8–12 weeks are sufficient to improve aerobic capacity, mitochondrial enzyme activity, and muscle function in MM patients (32, 45). Exercise physiology research has established that 6–8 weeks of training are generally required to achieve significant improvements in mitochondrial function and aerobic capacity; however, such adaptive changes gradually diminish after training cessation (89). This decline is primarily attributable to the limited adaptive reserve of MM patients, as their inherent mutation burden weakens compensatory capacity for energy metabolism. Consequently, the benefits of short-term training wane after discontinuation, highlighting the need for long-term or regular exercise regimens to sustain therapeutic effects.

In terms of frequency and duration, most studies adopted 3–5 sessions per week, each lasting 30–60 min (Table 4). Current evidence suggests that this regimen is both acceptable and effective for most MM phenotypes. It provides sufficient stimulus to drive mitochondrial adaptations while accounting for the limited physical capacity of MM patients. Excessive frequency or duration may reduce adherence, whereas insufficient training may fail to sustain benefits.

Low-intensity aerobic exercise can promote mitochondrial biogenesis and increase the number of mitochondria, although the extent of these changes is relatively modest (90). Moderate- and high-intensity aerobic exercise can more effectively stimulate mitochondrial biogenesis, achieve the greatest improvements in mitochondrial function (22, 91), and increase the lactate threshold. In addition, high-intensity exercise can enhance glycogen storage capacity (92), increase VO_2_ max and the upper limit of physical performance, and promote muscle hypertrophy by increasing muscle volume and maximal cross-sectional area (93). However, prolonged exercise at high intensity can lead to oxidative stress and muscle damage.

As mentioned earlier, resistance exercise, regardless of intensity, can increase muscle mass and strength. However, low-intensity resistance exercise is more similar to aerobic exercise and can promote mitochondrial biogenesis. Moderate-intensity resistance exercise can simultaneously enhance muscle mass and strength while improving mitochondrial function (26). High-intensity resistance exercise can further enhance the maximal rate of anaerobic glycolysis and improve the storage and regeneration capacity of intramuscular phosphocreatine and glycogen (94).

In terms of intensity, aerobic exercise at 60%–80% VO_2_ max/HRmax improves oxidative metabolism in MM patients without increasing CK levels or mutation burden. Similarly, resistance exercise at 70%–80% 1RM enhances muscle strength and reduces the proportion of COX-deficient fibers, also without elevating CK levels or mutation burden. These findings underscore the value of moderate-to-high intensity within a safe range, enabling patients to obtain exercise benefits while minimizing the risk of excessive metabolic stress.

Based on current research findings, although most studies have not observed a significant elevation in CK levels, MM patients may still experience exercise-induced muscle injury and face a potential risk of RML. Moderate-intensity aerobic exercise is generally well tolerated (33, 42, 43, 48, 49, 54); however, when training intensity is further increased, or when exercise frequency and duration exceed the individual recovery threshold, oxidative stress and metabolic load may rise markedly, exacerbating disease severity and increasing the likelihood of RML (35, 36, 95). Moderate-intensity resistance training also shows good tolerance, but when the load surpasses individual capacity, particularly during eccentric contractions, exhaustive training, or insufficient recovery, CK levels may increase, leading to skeletal muscle damage and, in severe cases, RML. Given the pronounced genotypic and phenotypic heterogeneity among MM patients, exercise tolerance varies considerably. Therefore, individualized adjustments to exercise intensity should be made based on functional capacity, mtDNA mutation burden, recovery response, and dynamic CK monitoring. Future studies should employ large-sample, phenotype-stratified clinical designs to identify safe training thresholds and risk indicators across different subtypes and mutation severities, thereby providing a more precise basis for developing exercise prescriptions for MM patients.

### 4.4 Phenotype-specific responses to exercise intervention

Existing studies demonstrate that exercise effects differ markedly across MM phenotypes (Table 5), indicating that adaptations are closely linked to genotype. Large-scale deletions and the m.3243A>G mutation respond favorably to both aerobic and resistance exercise (33, 45, 48, 51), whereas other point mutations, microdeletions, and rare genotypes show variable or even adverse responses (42, 44, 49). These discrepancies may stem from the impact of mutation sites on ETC complexes, the proportion of residual normal mtDNA, and heteroplasmy levels. Patients with large-scale deletions retain partial mitochondrial function, allowing training-induced improvements to translate into stable benefits. By contrast, mutations directly affecting key ETC subunits, such as Cyt b or COX I, may restrict compensatory metabolic capacity and even lead to detrimental effects.

These findings underscore that exercise prescriptions must be individualized according to the phenotype and mutation burden of MM patients.

Based on current evidence, MM patients with large-scale deletions are recommended to perform combined training: aerobic exercise at 60%–80% VO_2_ max, 3–5 sessions/wk, 30–45 min/session, together with resistance training at 70%–85% 1RM, 3 sets of 6–8 reps, to improve mitochondrial function and muscle strength.

For MM patients with point mutations, although those with the m.3243A>G variant can improve aerobic capacity and metabolic tolerance, some studies have reported an upward trend in mutation burden during detraining, indicating unstable adaptations. Therefore, low-to-moderate intensity aerobic training (∼60% VO_2_ max, 25–40 min, 3–4 sessions/wk) is recommended, while resistance training should be approached with caution. For MM patients with other point mutations, exercise-induced improvements are inconsistent. Moderate-to-low intensity aerobic training is recommended to avoid excessive loading. Importantly, in patients with mutations directly affecting ETC complexes, such as Cyt b or COX I, only low-intensity aerobic exercise should be applied with caution.

For MM patients with microdeletions, exercise responses are inconsistent, and in some cases mutation burden has increased. Therefore, only low-intensity aerobic training (≤60% VO_2_ max, 20–30 min, 2–3 sessions/wk) should be applied, while high-intensity exercise should be avoided. For patients with unverified or suspected nuclear genotypes, further confirmation is required, and current evidence is insufficient to guide exercise prescription.

It is important to note that these exercise prescriptions are derived from small sample studies and must be applied with individualized evaluation and dynamic monitoring of metabolic indices to avoid exacerbating functional impairment or increasing mutation burden. Future studies with larger cohorts and molecular confirmation are needed to validate and refine these preliminary conclusions.

These phenotype-specific recommendations highlight the importance of individualized exercise prescription in clinical settings. Clinicians should prioritize moderate-intensity aerobic exercise as the foundational intervention, gradually integrating resistance training depending on patient tolerance and mutation burden. Continuous safety monitoring is essential during exercise programs, including regular tracking of serum CK, lactate, and other metabolic biomarkers, alongside symptom-based evaluation such as muscle pain or dark urine. This approach enables early detection of metabolic imbalance or potential RML, ensuring therapeutic efficacy while minimizing risk.

### 4.5 Exercise combined with pharmacological or nutritional intervention

**Figure 6:**
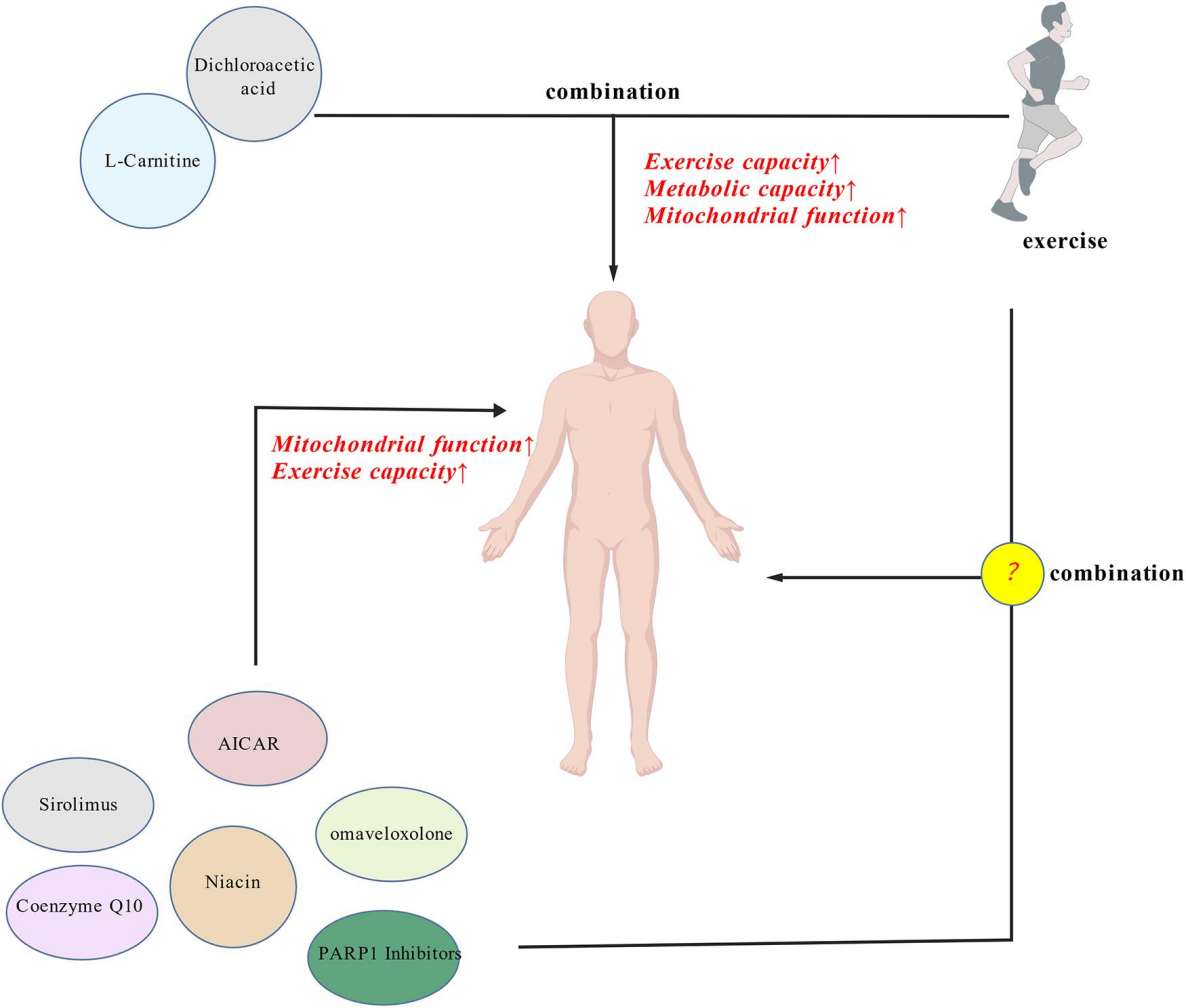
Synergistic effects of combined exercise and pharmacological or nutritional interventions on mitochondrial function and exercise capacity in mitochondrial myopathies. L-carnitine and dichloroacetate, as synergistic therapies, combined with exercise, can enhance exercise capacity, metabolic efficiency, and mitochondrial function. Other pharmacological interventions (such as AICAR, sirolimus, coenzyme Q10, niacin, omaveloxolone, and PARP1 inhibitors) show potential in improving mitochondrial function and exercise capacity, but their therapeutic effects when integrated with exercise require further investigation (Created with BioGDP.com; ID: GDP2025ZW3Q4E).

Some pharmacological strategies have been investigated to improve mitochondrial function in mitochondrial myopathy (MM), both in patients and in experimental models. Clinical studies in patients with MM have explored metabolic interventions such as dichloroacetate, L-carnitine, niacin, Coenzyme Q10 (CoQ10), and oligomycin derivatives (96–100). For example, the combination of dichloroacetate and L-carnitine with exercise has been reported to improve mitochondrial function and enhance aerobic metabolic capacity in MM patients. These findings suggest that pharmacological or nutritional support targeting mitochondrial metabolism may potentially complement exercise interventions. However, the effectiveness of integrating these agents into exercise prescriptions for MM patients remains to be validated (Figure 6) (20).

In addition to clinical observations in MM patients, several pharmacological strategies have also been investigated in experimental MM mouse models to explore their potential effects on mitochondrial function and muscle performance (101–103). Niacin has been reported to enhance mitochondrial biogenesis and improve metabolic capacity in both MM patients and experimental models (100, 102, 103). CoQ10, a key coenzyme in the electron transport chain, may improve mitochondrial oxidative phosphorylation efficiency and reduce oxidative stress (97). Oligomycin derivatives have been shown to reduce heart rate and lactate levels during submaximal exercise in MM patients and may contribute to improved mitochondrial function (99). In experimental MM mouse models, AICAR has been reported to enhance mitochondrial function and muscle performance while promoting muscle fiber regeneration (102). Similarly, PARP1 inhibitors improve exercise endurance and mitochondrial function in MM mouse models (101), and rapamycin has been shown to slow disease progression, maintain mitochondrial homeostasis, and reduce mtDNA mutation load in experimental models (104).

These metabolic effects may potentially complement exercise interventions and could also have implications for safety. Theoretically, nutritional supplements that support mitochondrial oxidative metabolism could help reduce exercise-induced muscle injury or RML risk by improving mitochondrial energy supply and attenuating ROS production. Evidence from related metabolic myopathies, such as glycogen storage diseases and fatty acid oxidation disorders, indicates that tailored nutritional strategies including pre-exercise carbohydrate supplementation and medium-chain triglyceride intake can mitigate exercise-induced energy crisis and reduce RML risk (105–109). Although these disorders differ fundamentally from mitochondrial myopathies in their primary metabolic defects, involving cytosolic glycolysis or beta-oxidation rather than mitochondrial oxidative phosphorylation, they share a similar vulnerability to exercise-induced energy depletion, which provides a useful comparative framework. However, clinical evidence remains limited, and future studies are needed to verify whether combined nutritional or pharmacological interventions can provide protective effects during exercise in patients with mitochondrial myopathy.

Based on existing research findings and the heterogeneity in genetic mutations and phenotypic responses among patients with MM, several potential phenotype-specific strategies combining exercise and metabolic support can be hypothesized to guide future research on individualized therapy.

For patients with large-scale deletions, a regimen combining moderate-intensity aerobic exercise with high-intensity resistance training, alongside supplementation with CoQ10 or dichloroacetate (DCA), may enhance mitochondrial oxidative metabolism and improve ADP clearance kinetics.

In cases involving point mutations, such as 3243A>G, low- to moderate-intensity aerobic training, combined with pharmacological agents like AICAR or nicotinic acid that activate the AMPK/PGC-1*α* pathway, may stimulate mitochondrial biogenesis and mitigate oxidative stress.

In cases involving microdeletions, Patients with rare microdeletions display variable or unstable exercise responses, with some evidence of increased mutation load following training. Very low-intensity aerobic training (≤60% VO_2_ max equivalent) or respiratory muscle exercise may be cautiously considered. In preclinical models, rapamycin has been shown to preserve mitochondrial homeostasis, suggesting potential as an adjunct (6).

These recommendations are provisional and derived from small-sample studies. In practice, individualized monitoring should be prioritized, with comprehensive evaluation of CK changes, recovery responses, and metabolic status to ensure that supplementation strategies enhance adaptation without increasing physiological burden. Future studies should further investigate, within the framework of combined pharmacological and exercise interventions, the potential regulatory effects of these strategies on RML risk.

### 4.6 Limitations and future directions

This review synthesized evidence on the effects of exercise in MM patients. Evidence from 15 studies showed that moderate-intensity, progressively structured interventions improved VO_2_ max, muscle strength, and enzymatic activity without increasing mutation burden or CK levels. Patients were categorized by mutation type into subgroups, and mutation-specific exercise recommendations were proposed. In addition, the potential of combining exercise with supplements such as CoQ10, DCA, and niacin was explored, offering perspectives for integrative therapeutic models.

This review has several limitations. The number of included studies was limited, most being small-sample and short-term clinical trials, and the lack of large-scale, robust RCTs restricts the strength of the conclusions. In addition, variations in exercise type, intensity, frequency, and duration across studies introduced substantial heterogeneity, hindering the development of standardized, broadly applicable exercise guidelines. Given the heterogeneity among MM patients, specific phenotypes often exhibit weak or unstable responses to exercise, which may in some cases pose potential risks. To date, most studies have been limited to short-term interventions, with a lack of systematic longitudinal research to assess the long-term progression of mutation burden, cardiac stress, and multi-organ involvement. Furthermore, the inclusion of only English-language studies may have led to language bias, and the possibility of publication bias cannot be ruled out, as studies with negative or null results are less likely to be published.

Another key limitation is the limited evaluation of exercise-related safety outcomes, particularly the risk of exercise-induced RML. Although most included studies did not report significant increases in CK levels, systematic assessment of RML incidence, severity, and recovery under different training intensities and modalities remains insufficient. In addition, it is still unclear whether pharmacological or nutritional interventions, such as CoQ10, niacin, AICAR, or L-carnitine, can help reduce the risk of exercise-associated RML in MM patients. Future research should include standardized biochemical safety monitoring and detailed reporting of adverse events to better define the safety profile of exercise-based interventions in this population.

Future research should aim to conduct large-scale RCTs to clarify the causal and dose–response relationships between different exercise modalities and MM phenotypic responses. Future efforts should also focus on developing precise stratification strategies that account for mutation type, mutation burden, and the degree of multi-organ involvement, in order to optimize personalized exercise prescriptions. In addition, the mechanisms and safety of combined interventions integrating exercise with supplements such as CoQ10, DCA, and niacin should be verified to support comprehensive therapeutic development. Long-term monitoring systems should be established by incorporating mutation burden tracking, cardiotoxicity surveillance, organ function assessment, and quality-of-life measures, in order to evaluate the sustained efficacy and potential risks of such interventions.

## 5 Conclusions

Evidence from both clinical and experimental studies indicates that aerobic exercise, resistance training, or their combination can enhance mitochondrial function, muscle strength, and metabolic capacity in MM patients without increasing mutation burden or CK levels. Exercise prescriptions should be developed based on clinical phenotypic patterns, such as single large-scale deletions or point mutation-related subtypes, to ensure safety and effectiveness. Compared to acute or high-intensity interventions, long-term and progressively adjusted training regimens appear more beneficial in promoting mitochondrial remodeling and minimizing metabolic stress. Combining exercise with agents like CoQ10, DCA, or niacin may enhance outcomes, their safety and efficacy across specific MM phenotypes remain to be validated in future.

## Data Availability

The original contributions presented in the study are included in the article/Supplementary Material, further inquiries can be directed to the corresponding author.
