# Supplementary material for: Effects of exercise training on skeletal muscle function in patients with mitochondrial myopathy: a systematic review
Source: Front Sports Act Living. 2026 Apr 9;8:1710264. doi: 10.3389/fspor.2026.1710264 (PMC13102759; doi:10.3389/fspor.2026.1710264)
Supplement: Supplementary file 1 [file Datasheet1.docx]

# Supplementary File 1. Search Terms

**PubMed:**

( "Mitochondrial Myopathies"[MeSH] OR "mitochondrial myopathy"[tiab] OR "mitochondrial myopathies"[tiab] OR "mitochondrial disease"[tiab] OR "mtDNA defect*"[tiab] OR "metabolic myopathy"[tiab] ) AND ( "Exercise Therapy"[MeSH] OR "Exercise"[MeSH] OR exercise[tiab] OR training[tiab] OR "physical activity"[tiab] OR aerobic[tiab] OR endurance[tiab] OR resistance[tiab] OR strength[tiab] OR rehabilitation[tiab] ) AND ( trial[tiab] OR intervention[tiab] OR "randomized controlled trial"[pt] OR "clinical trial"[pt] ) AND (human[MeSH]) AND ("1990/01/01"[dp] : "2025/09/01"[dp])

**Web of science:**

TS=("mitochondrial myopathy" OR "mitochondrial myopathies" OR "mitochondrial disease" OR "mtDNA defect" OR "metabolic myopathy" OR "metabolic myopathies") AND TS= ("Exercise Therapy" OR exercise OR training OR "physical activity" OR aerobic OR endurance OR resistance OR strength OR rehabilitation) AND TS= (trial OR intervention OR randomized OR conditioning OR "exercise program" OR "exercise therapy" OR "exercise capacity") AND PY=1990-2025 AND DT=Article AND LA=English

**Embase:**

('mitochondrial myopathy'/exp OR 'mitochondrial myopathy':ti,ab OR 'mitochondrial myopathies':ti,ab OR 'mitochondrial disease':ti,ab OR 'mtdna defect*':ti,ab OR 'metabolic myopathy':ti,ab OR 'metabolic myopathies':ti,ab) AND ('exercise therapy'/exp OR 'physical training'/exp OR 'exercise therapy':ti,ab OR exercise:ti,ab OR training:ti,ab OR 'physical activity':ti,ab OR aerobic:ti,ab OR endurance:ti,ab OR resistance:ti,ab OR strength:ti,ab OR rehabilitation:ti,ab) AND (trial:ti,ab OR intervention:ti,ab OR randomized:ti,ab OR conditioning:ti,ab OR 'exercise program':ti,ab OR 'exercise therapy':ti,ab OR 'exercise capacity':ti,ab) AND [1990-2025]/py AND [english]/lim NOT ('animal'/exp NOT 'human'/exp) NOT 'review'/it

**Scopus:**

TITLE-ABS-KEY ( "mitochondrial myopathy" OR "mitochondrial myopathies" OR "mitochondrial disease" OR "mtDNA defect*" OR "metabolic myopathy" OR "metabolic myopathies" ) AND TITLE-ABS-KEY ( "Exercise Therapy" OR exercise OR training OR "physical activity" OR aerobic OR endurance OR resistance OR strength OR rehabilitation ) AND TITLE-ABS-KEY ( trial OR intervention OR randomized OR conditioning OR "exercise program" OR "exercise therapy" OR "exercise capacity" ) AND ( PUBYEAR > 1989 AND PUBYEAR < 2026 ) AND ( LIMIT-TO ( DOCTYPE , "ar" ) ) AND ( LIMIT-TO ( LANGUAGE , "English" ) )

**Supplementary Table S1. Oxygen uptake outcomes reported in the included studies.**

| **Type of exercise** | **Exercise intensity** | **Sample** | **Oxygen uptake** | **Baseline** | **Post training** |
| --- | --- | --- | --- | --- | --- |
| Aerobic Exercise | 70%-80% HR max  3-4×/wk | n=8  large-scale mtDNA deletions  (Taivassalo et al., 2006) | Peak VO₂ (L/min) | 1.36 ± 0.4 | 1.51 ± 0.4* (↑ 11%) |
| Aerobic Exercise | 65-75% VO_2_max  50 sessions/12 wk | n=20  5 single large-scale deletions  1 microdeletion  14-point mutations  (Jeppesen et al., 2006) | VO_2_ max (ml·kg⁻¹·min⁻¹) | 25.1 ± 2.5 | 32.0 ± 3.0* (↑ 26%) |
| Aerobic Exercise | MHR 65%-70%  4×/wk, 30-45min | n=6  Genotype not specified  (Porcelli et al., 2016) | Peak VO₂(ml·kg⁻¹·min⁻¹) | 14.7 ± 1.2 | 17.6 ± 1.4* (↑ 19.7%) |
| Aerobic Exercise | MHR 70-80%  3-4×/wk, 30→40 min | n=10  2 single large-scale deletions  2 tRNA mutations  1 ND4 mutation  2 Cyt b mutations  1 COX I mutation  1 COX III microdeletion  1 uncharacterized  (Taivassalo et al., 2001) | Peak VO₂ (ml·kg⁻¹·min⁻¹) | 13.0 ± 5.1 | 15.3 ± 5.5* (↑ 17.7%) |
| Combined exercise | Aerobic exercise (8wk)  Intensity: 65%→75%→85%→90→100% PPO  Resistance training (8wk) Intensity: RPE 6-7/10  3×/wk, 60-90 min  Inspiratory muscle training (8wk)  Threshold device at 40% PI max  2×/day × 30 breaths | n=12  4 single large-scale deletions  5 multiple deletions  3-point mutations  (Fiuza-Luces et al., 2018) | Peak VO₂ (mL·kg⁻¹·min⁻¹) | 22.1 ± 1.8 | 25.8 ± 1.8* (↑ 16.7%) |
| Combined exercise | Aerobic Exercise + Resistance training (12wk)  70% max workload / 50%  1RM 3×/wk, 1h | n=18  Genotype not specified  (Cejudo et al., 2005) | VO₂ max (L/min) | 1.4 ± 0.4 | 1.8 ± 0.5* (↑ 28%) |

wk: week; min: minute; HR max: maximal heart rate; MHR: maximal heart rate; VO₂ max: maximal oxygen uptake; Peak VO₂: peak oxygen uptake; PPO: peak power output; RPE: rating of perceived exertion; 1RM: one repetition maximum; PImax: maximal inspiratory pressure; mtDNA: mitochondrial DNA; tRNA: transfer RNA; ND4: NADH dehydrogenase subunit 4; Cyt b: cytochrome b; COX I: cytochrome c oxidase subunit I; COX III: cytochrome c oxidase subunit III. Values are presented as mean ± SD. * *p* < 0.05 (vs baseline); ↑: increase compared with baseline; ↓: decrease compared with baseline.

**Supplementary Table S2. Power output outcomes reported in the included studies.**

| **Type of exercise** | **Exercise intensity** | **Sample** | **Power output** | **Baseline** | **Post training** |
| --- | --- | --- | --- | --- | --- |
| Aerobic exercise | 70%-80% HR max  3-4×/wk | n=8  large-scale mtDNA deletions  (Taivassalo et al., 2006) | Peak Watts (W) | 85.6 ± 26 | 107.5 ± 32* (↑ 26%) |
| Aerobic exercise | 65-75% VO_2_max  50 sessions/12 wk | n=20  5 single large-scale deletions  1 microdeletion  14-point mutations  (Jeppesen et al., 2006) | W max (W) | 102.4 ± 10.9 | 131.2 ± 15.6* (↑ 29%) |
| Aerobic exercise | MHR 65%-70%  4×/wk, 30-45min | n=6  Genotype not specified  (Porcelli et al., 2016) | Peak Work Rate (W) | 72 ± 13 | 88 ± 15* (↑ 22.2%) |
| Aerobic exercise | MHR 70-80%  3-4×/wk, 30→40 min | n=10  2 single large-scale deletions  2 tRNA mutations  1 ND4 mutation  2 Cyt b mutations  1 COX I mutation  1 COX III microdeletion  1 uncharacterized  (Taivassalo et al., 2001) | Peak Watts (W) | 45 ± 21 | 59 ± 27* (↑ 31.1%) |
| Aerobic exercise | MHR 70-80%  3×/wk, 30 min | n=10  5-point mutation (m.3243A>G)  5 ragged-red fibers  (Trenell, 2006) | Peak Watts (W) | 49 ± 19 | 53 ± 20* (↑ 8.2%) |
| Aerobic exercise | Anaerobic threshold intensity  3×/wk, 30–45 min | n=10  10 m.3243A>G  (Bates, 2013) | PPO (W) | 100 ± 45 | 112 ± 55* (↑ 12.0%) |
| Combined exercise | Aerobic exercise (8wk)  Intensity: 65%→75%→85%→90→100% PPO  Resistance training (8wk) Intensity: RPE 6-7/10  3×/wk, 60-90 min  Inspiratory muscle training (8wk)  Threshold device at 40% PI max  2×/day × 30 breaths | n=12  4 single large-scale deletions  5 multiple deletions  3-point mutations  (Fiuza-Luces et al., 2018) | PPO (W) | 98 ± 11 | 123 ± 13* (↑ 25.5%) |
| Combined exercise | Aerobic Exercise + Resistance training (12wk)  70% max workload / 50%  1RM 3×/wk, 1h | n=18  Genotype not specified  (Cejudo et al., 2005) | W max (W) | 58 ± 25 | 67 ± 20* (↑ 15.5%) |

wk: week; min: minute; HR max: maximal heart rate; MHR: maximal heart rate; VO₂ max: maximal oxygen uptake; W max: maximal workload; Peak Watts: peak power output; Peak Work rate: peak exercise work rate; PPO: peak power output; RPE: rating of perceived exertion; 1RM: one repetition maximum; PImax: maximal inspiratory pressure; mtDNA: mitochondrial DNA; tRNA: transfer RNA; ND4: NADH dehydrogenase subunit 4; Cyt b: cytochrome b; COX I: cytochrome c oxidase subunit I; COX III: cytochrome c oxidase subunit III. Values are presented as mean ± SD; * *p* < 0.05 (vs baseline); ↑ increase compared with baseline; ↓ decrease compared with baseline.

**Supplementary Table S3. Lactate outcomes reported in the included studies.**

| **Type of exercise** | **Exercise intensity** | **Sample** | **Lactate** | **Baseline** | **Post training** |
| --- | --- | --- | --- | --- | --- |
| Aerobic Exercise | 70%-80% Hrmax  3-4×/wk | n=8  large-scale mtDNA deletions  (Taivassalo et al., 2006) | Lactate (mmol/L⁻¹) | 3.5 ± 0.7 | 2.2 ± 0.6* (↓ 37%) |
| Aerobic Exercise | 65-75% VO2max  50 sessions/12 wk | n=20  5 single large-scale deletions  1 microdeletion  14-point mutations  (Jeppesen et al., 2006) | Lactate (mmol/L⁻¹) | 8.7 ± 3.2 | 9.0 ± 3.0* (↑ 3.4%) |
| Aerobic Exercise | near-LT workload (~40-50% pnPOmax)  3-4×/wk, 30-45 min | n=12  6 single large-scale deletions  2 uncharacterized  3 multiple deletions  1 point mutation  (Siciliano, 2000) | Lactate (mmol/L⁻¹) | 4.50 ± 0.61 | 2.76 ± 0.54* (↓ 38.7%) |
| Aerobic Exercise | HRR 60%-80%  3-4×/wk, 20-30min | n=10  5 deletions  2-point mutations  3 uncharacterized mtDNA (2 suspected nuclear mutations)  (Taivassalo, 1998) | Blood lactate at rest (mmol/L⁻¹)  Blood lactate after exercise (mmol/L⁻¹) | 3.27 ± 1.27  4.78 ± 1.66 | 2.30 ± 0.98* (↓ 29.7%)  3.19 ± 2.23* (↓ 33.3%) |
| Aerobic Exercise | 40% pnPOmax,  30→45min | n=7  4 single large-scale deletions  3 multiple deletions  (Siciliano, 2012) | Lactate (mmol·L⁻¹) | 6.34 ± 2.56 | 3.97 ± 1.73 (↓ 37.4%) |
| Aerobic Exercise | HRR 70%-85%  3–4×/wk, 20-30 min | n=14  9 single large-scale deletions  2-point mutations  3 unclassified  (Taivassalo, 1999) | Rest lactate (mmol/L⁻¹)  Post-exercise lactate (mmol/L⁻¹) | 3.26 ± 1.13  4.7 ± 1.6 | 2.46 ± 0.85* (↓ 24.5%)  3.4 ± 2.0* (↓ 27.7%) |

wk: week; min: minute; HR max: maximal heart rate; HRR: heart rate reserve; MHR: maximal heart rate; VO₂ max: maximal oxygen uptake; LT: lactate threshold; pnPOmax: peak power output predicted from maximal incremental test; PPO: peak power output; RPE: rating of perceived exertion; 1RM: one repetition maximum; PImax: maximal inspiratory pressure; mtDNA: mitochondrial DNA; tRNA: transfer RNA; ND4: NADH dehydrogenase subunit 4; Cyt b: cytochrome b; COX I: cytochrome c oxidase subunit I; COX III: cytochrome c oxidase subunit III; Values are presented as mean ± SD; * *p* < 0.05 (vs baseline); ↑: increase compared with baseline; ↓: decrease compared with baseline.

**Supplementary Table S4. Heart rate outcomes reported in the included studies.**

| **Type of exercise** | **Exercise intensity** | **Sample** | **Heart rate** | **Baseline** | **Post training** |
| --- | --- | --- | --- | --- | --- |
| Aerobic exercise | 70%-80% Hrmax  3-4×/wk | n=8  large-scale mtDNA deletions  (Taivassalo et al., 2006) | HR (bpm) | 141 ± 17 | 123 ± 16* (↓ 13%) |
| Aerobic exercise | 65-75% VO_2_max  50 sessions/12 wk | n=20  5 single large-scale deletions  1 microdeletion  14-point mutations  (Jeppesen et al., 2006) | HR (bpm) | 166 ± 18 | 165 ± 18* (↓ 0.6%) |
| Aerobic exercise | near-LT workload (~40-50% pnPOmax)  3-4×/wk, 30-45 min | n=12  6 single large-scale deletions  2 uncharacterized  3 multiple deletions  1 point mutation  (Siciliano, 2000) | HR (bpm) | 116.4 ± 2.2 | 109.6 ± 1.9 (↓ 5.8%) |
| Aerobic exercise | HRR 60%-80%  3-4×/wk, 20-30min | n=10  5 deletions  2-point mutations  3 uncharacterized mtDNA (2 suspected nuclear mutations)  (Taivassalo, 1998) | HR at rest (bpm)  HR after exercise (bpm) | 99.11 ± 9.92  161.44 ± 9.88 | 88.78 ± 11.64* (↓ 10.4%)  140.33 ± 11.05* (↓ 13.1%) |
| Aerobic exercise | HRR 70%-85%  3–4×/wk, 20-30 min | n=14  9 single large-scale deletions  2-point mutations  3 unclassified  (Taivassalo, 1999) | HR (bpm) | 162 ± 9 | 141 ± 13* (↓ 13.0%) |

wk: week; min: minute; HR: heart rate; HR max: maximal heart rate; HRR: heart rate reserve; VO₂ max: maximal oxygen uptake; LT: lactate threshold; pnPOmax: peak power output predicted from maximal incremental test; mtDNA: mitochondrial DNA. Values are presented as mean ± SD. * *p* < 0.05 (vs baseline); ↑: increase compared with baseline; ↓: decrease compared with baseline.
